# A new species of the genus *Himertosoma* from the Ryukyus, Japan, with a key to species from the Palaearctic and Oriental Regions (Hymenoptera, Ichneumonidae, Banchinae)

**DOI:** 10.3897/zookeys.234.3794

**Published:** 2012-10-30

**Authors:** Kyohei Watanabe, Kaoru Maeto

**Affiliations:** 1 Laboratory of Insect Biodiversity and Ecosystem Science, Graduate School of Agricultural Science, Kobe University, Rokkodaicho 1–1, Nada, Kobe, Hyogo 657–8501, Japan

**Keywords:** Amamioshima, Atrophini, endemic species, fauna, taxonomy

## Abstract

A new species of the genus *Himertosoma* Schmiedeknecht, *Himertosoma kuslitzkii* **sp. n.**, was discovered in Amamioshima Island, the Ryukyus. This new species resembles two Oriental species, *Himertosoma philippense* Chandra & Gupta and *Himertosoma townesi* Chandra & Gupta, in the colour pattern of the head and metasoma, number of flagellomeres, and the relatively slender first metasomal tergite, but can easily be distinguished from them by the nearly evenly punctate propodeum, different length/width ratio of the first metasomal tergite, different length of the ovipositor sheath, tricoloured mesosoma, and the whitish band along the posterior margin of the second and following metasomal tergites. A key to the Palaearctic and Oriental species of *Himertosoma* is also provided.

## Introduction

*Himertosoma* Schmiedeknecht, 1900, is a large genus in the tribe Atrophini of the ichneumonid subfamily Banchinae, containing 58 described species (Yu et al. 2012). The genus is found in the Ethiopian, Palearctic and Oriental Regions, but its species richness is strongly biased to the Ethiopian Region. Only four species have been known from the Palearctic and Oriental Regions; *Himertosoma superbum* Schmiedeknecht, 1900 (type species) from Egypt, Israel, Syria and Uzbekistan, *Himertosoma uchidai* Kuslitzky, 2007 (= *Himertosoma sulcata* Kuslitzky, 1995) from Far East Russia and Japan (Kunashiri Island), and *Himertosoma philippense* Chandra & Gupta, 1977 and *Himertosoma townesi* Chandra & Gupta, 1977 from the Philippines.

The host records are from three families of Lepidoptera, i.e., Gelechiidae (*Amblypalpis tamaricella* Danilevsky and *Parapodia tamaricicola* Joannis) and Yponomentidae (*Prays oleae* Bernard) for *Himertosoma superbum*, and Tortricidae (*Cydia pomonella* (Linnaeus)) for *Himertosoma stramineum* (Morley) (Yu et al. 2012). Although details of the biology are unknown for *Himertosoma*, other members of Banchinae are exclusively koinobiont endoparasitoids (Wahl 1993).

In addition to *Himertosoma uchidai* from Kunashiri Is. (Kuslitzky 2007), we found a species of *Himertosoma* from Japan, collected on Amamioshima Island, the Ryukyus, which is described here as new to science. A key to all the described species of *Himertosoma* from the Palaearctic and Oriental Regions is also provided.

In this paper, we describe the new species based on only a single specimen, for the three following reasons: 1) the specimen is in good condition, 2) we made intensive collecting efforts in the type locality and checked all major collections of Japanese ichneumonids, but we have not found any more specimens, and 3) we know that the ichneumonid fauna of the Ryukyus contains some biogeographically important species such as relict or endemic species (e.g., Watanabe et al. 2010; Matsumoto and Broad 2011).

## Materials and methods

A stereomicroscope (Nikon S800) was used for observation. Digital images were edited using Adobe Photoshop® CS3. Terminology for general morphology follows Gauld (1991) and for surface sculpture follows Eady (1968). The methods used for measuring clypeus, face, malar space (= cheek of Townes) and metasomal tergites follow Townes (1969). The type specimen of the new species will be deposited in the National Institute for Agro–Environmental Sciences, Tsukuba, Ibaraki, Japan. The holotypes of two Oriental species, *Himertosoma philippense* (♀) and *Himertosoma townesi* (♀), deposited in the American Entomological Institute, were also examined for comparison. The character states of two Palaearctic species, *Himertosoma superbum* and *Himertosoma uchidai*, are taken from the descriptions by Schmiedeknecht (1907) and Kuslitzky (1995), respectively. Characteristics of *Himertosoma superbum* are also available from the excellent figure in Townes (1970).

## Taxonomy

### Genus Himertosoma

Schmiedeknecht, 1900

http://species-id.net/wiki/Himertosoma

Himertosoma Yu and Horstmann (1997) for synonymy.

#### Remarks.

According to Townes (1970) and Chandra and Gupta (1977), this genus can be separated from other atrophine genera by the combination of the following character states: occipital carina complete, its lower end joining hypostomal carina; mesoscutum often smooth; areolet always absent; tarsal claws sometimes only partly pectinate; first metasomal tergite covered with longitudinal striation, with more or less distinct median dorsal carina basally, spiracle in front of middle (Figs 7, 8); laterotergite of fifth metasomal tergite not separated by a crease; exposed portion of fifth metasomal tergite of female only about 0.5 times as long as exposed portion of fourth metasomal tergite; and ovipositor distinctly longer than hind tibia (more than 1.4 times as long as hind tibia). However, the classification of *Himertosoma* and its most similar genus, *Lissonota* Gravenhorst, is still in dispute; *Himertosoma* can be separated from *Lissonota* only by a single character, the absence of the crease separating laterotergite of the fifth metasomal tergite. Their generic status should be reconsidered in future study.

The genus *Himertosoma*, hitherto known only from the Philippines in the Oriental Region (Chandra and Gupta 1977), was discovered for the first time on Amamioshima Island, the North Ryukyus, represented by the following new species.

### Himertosoma kuslitzkii sp. n.

urn:lsid:zoobank.org:act:DAF3559C-EB4B-4376-8EB5-E0348ED4523E

http://species-id.net/wiki/Himertosoma_kuslitzkii

Figs 1 –9

#### Type specimen.

Holotype: ♀, Japan, Kagoshima Pref., Amamioshima Island, Sumiyou Village, near Santaro–toge, 4. June 2007, Kyohei Watanabe leg.

#### Description.

Body length 6.5 mm; length of fore wing 4.5 mm. Head 0.6 times as long as wide in dorsal view; clypeus 0.7 times as long as wide, smooth excluding some punctures along weak supraclypeal suture, its profile gently convex in lateral view (Fig. 4); face 0.6 times as long as wide, median part longitudinally weakly convex, area excluding this convexity and below antennal socket covered with dense punctures (Figs 3, 4); frons sparsely punctate (Fig. 3); malar space 1.0 times as long as basal width of mandible; base of mandible evenly and slightly convex; lower tooth of mandible slightly shorter than upper one; vertex, gena and occiput smooth (but with minute setae and their sockets), excluding oceller area with some punctures and minute rugulae; minimum distance between lateral ocellus and margin of eye (OOL) 1.4 times as long as maximum diameter of lateral ocellus; minimum distance between lateral ocelli (POL) 2.0 times as long as maximum diameter of lateral ocellus. Antenna with 31 flagellomeres; first flagellomere 6.7 times as long as apical width and 1.3 times as long as second flagellomere.

Mesosoma 2.9 times as long as minimum distance between tegulae in dorsal view, polished, densely punctate excluding postero–lateral area of pronotum, mesonotum and postscutellum; epomia indistinct, obscurely present on collar (Fig. 5); mesoscutum smooth excluding anterior face, along lateral margin and notauli, and postero–median area sparsely punctate (Fig. 5); scutellum slightly convex, covered with sparse punctures; upper part of epicnemial carina nearly straight, reaching lower 1/4 of pronotum (Fig. 5); episternal scrobe small, narrowly smooth; propodeum with complete posterior transverse carina and pleural carina, with area petiolaris broadly smooth medially (Fig. 6); propodeal spiracle round. Legs: hind femur 6.0 times as long as deep, slightly bulged ventrally near base; hind tibia 9.5 times as long as wide; hind first tarsomere 2.1 times as long as second hind tarsomere and 3.0 times as long as longer hind tibial spur; tarsal claws entirely pectinate. Wings: fore wing with Cu–a distant from vein Rs+M by 0.6 times length of vein Cu–a; areolet absent; hind wing with distal abscissa of vein Cu1 much closer to vein 1A than to vein M, basal abscissa of vein Cu1 5.0 times as long as length of vein cu–a.

Metasoma polished and slender; first tergite 1.7 times as long as maximum width, 1.1 times as long as second tergite, densely longitudinally striate and sparsely punctate (Figs 7, 8); second tergite 1.1 times as long as maximum width; second to fourth tergites covered with dense, large punctures excluding smooth area along each posterior margin (Figs 2, 7, 8); laterotergite of fifth tergite absent (Fig. 9); fifth and following tergites alutaceous with fine, sparse punctures; ovipositor sheath 3.0 times as long as hind tibia and 1.2 times as long as fore wing.

**Figures 1–2.**
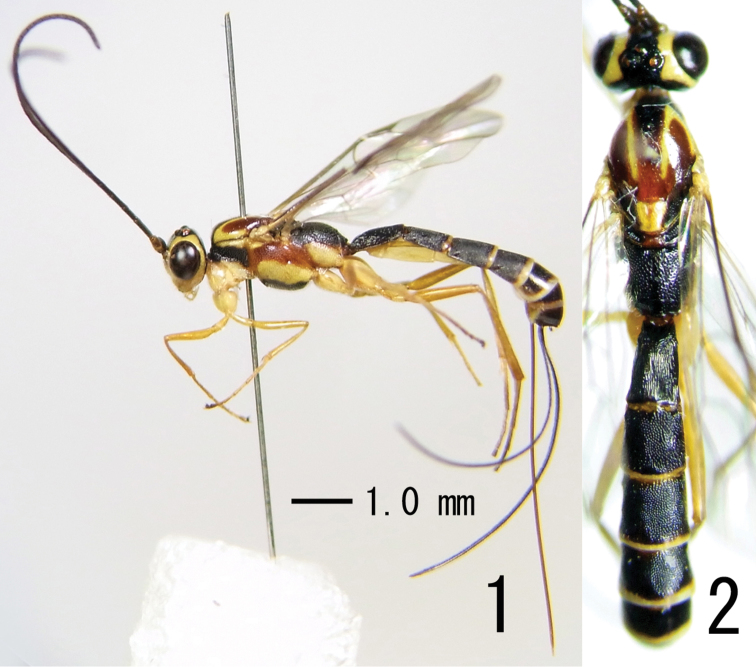
*Himertosoma kuslitzkii* sp. n., female (holotype) **1** Body, lateral view **2** head, mesosoma and metasoma, dorsal view.

**Figures 3–9.**
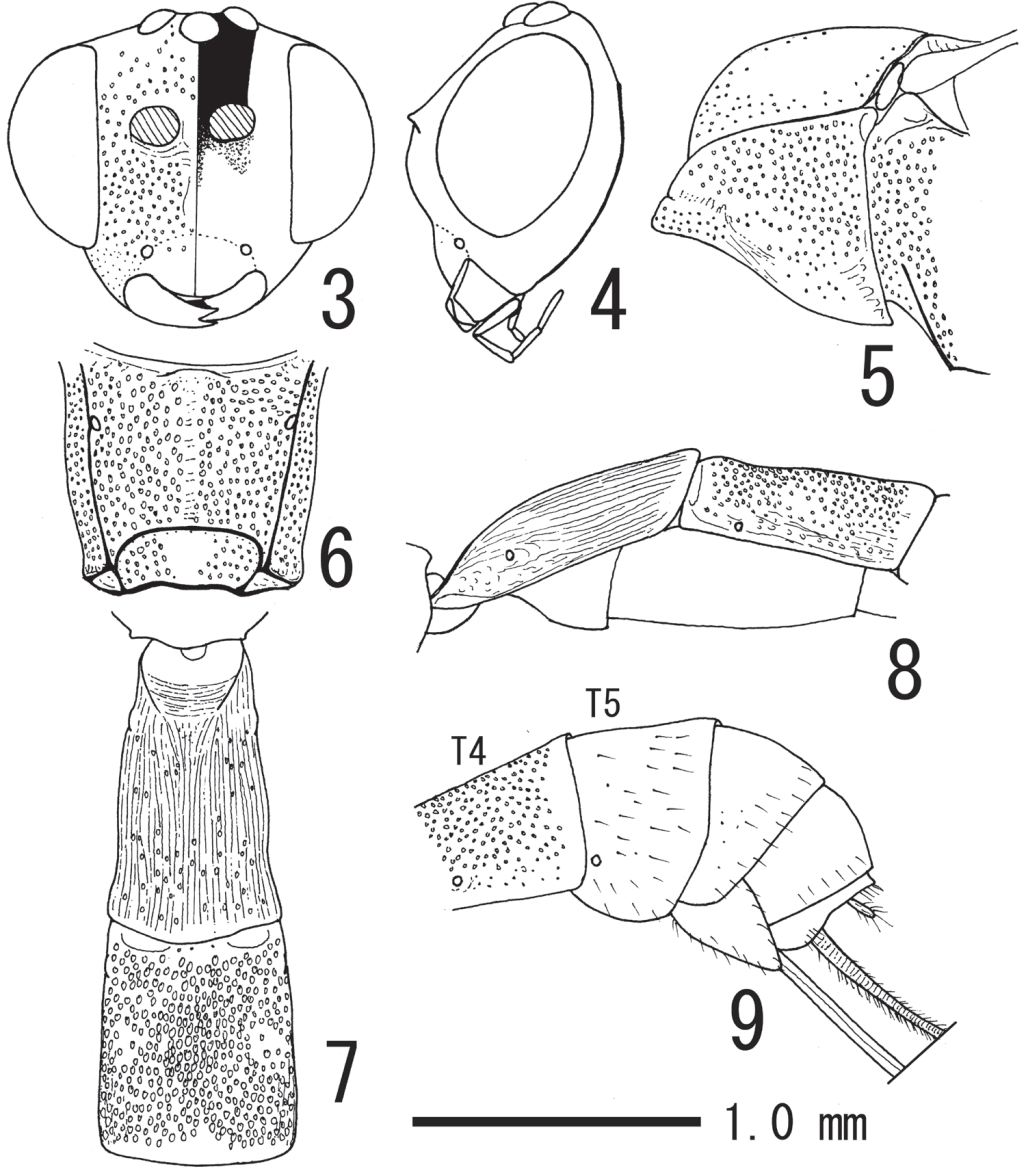
*Himertosoma kuslitzkii* sp. n., female (holotype) **3, 4** head, frontal (**3** right half indicates colour pattern) and lateral (**4** sculpture omitted) views; **5** anterior part of mesothorax, lateral view **6** propodeum, dorsal view **7, 8** first and second metasomal tergites, dorsal (**7**) and lateral (**8** punctation omitted on first metasomal tergite) views; **9** apical part of metasoma, lateral view.

Colouration (Figs 1–3). Head yellow except for: scape and pedicel brown; apex of mandible, antenna excluding scape and pedicel, longitudinal stripes below antennal sockets, frons, vertex and gena excluding orbit, occiput black. Mesosoma black, except for lateral longitudinal spots along upper and lower margins of propleuron, four longitudinal stripes on mesoscutum, two of these stripe in both sides connected anteriorly, scutellum excluding median reddish longitudinal area, tegula, subalar prominence, lower part of mesopleuron, posterior part of metapleuron yellow; median and lateral lobes on mesoscutum excluding yellowish stripe and anterior black area on median lobe of mesoscutum, mesopleuron excluding yellow area, anterior part of metapleuron red. Legs yellow, except for: ventral surface of hind coxa, hind femur, hind tibia, hind tarsus slightly brownish. Wings hyaline. Metasoma black, except for: membranous parts of sternites yellow; posterior margin of second and following tergites, posterior margin of subgenital plate whitish-yellow; subgenital plate excluding white posterior margin, ovipositor brown.

**Male.** Unknown.

#### Distribution.

Japan (Ryukyus: Amamioshima Island).

#### Etymology.

This species is named after V. S. Kuslitzky, who has contributed to the classification of Banchinae with excellent observations and to the faunal knowledge of ichneumonids in Far East Asia.

#### Remarks.

This species has all the characteristics of *Himertosoma* mentioned above and is distinguished from congeners by the combination of the following character states: flagellum with around 31 segments; propodeum nearly evenly punctate in front of posterior transverse carina (Fig. 6); first metasomal tergite 1.7 times as long as maximum width; ovipositor sheath 3.0 times as long as hind tibia; mesosoma tricoloured (black, yellow and red) (Figs 1, 2); second and following metasomal tergites with whitish bands along posterior margins (Figs 1, 2); ovipositor sheath 3.0 times as long as hind tibia and 1.2 times as long as fore wing; and mesosoma tricoloured.

This species resembles two Oriental species, *Himertosoma philippense* and *Himertosoma townesi*, in having similar colour patterns on the head and metasoma, the over 30–segmented flagellum and the relatively long first metasomal tergite, but can be easily distinguished from them as shown in the following key.

##### Key to Palaearctic and Oriental species of *Himertosoma* (♀)

**Table:** 

1	First metasomal tergite wide, 1.0–1.4 times as long as maximum (apical) width. Antenna with 26–27 flagellomeres. Inner orbit without yellow marking (face completely black) or metasomal tergites tinged with red. Palaearctic Region	2
–	First metasomal tergite slender, 1.7–2.0 times as long as maximum width. Antenna with 30–32 flagellomeres. Inner orbit with yellow stripe or face completely yellow. Metasomal tergites largely black, without conspicuous reddish areas. Oriental Region	3
2	First metasomal tergite 1.4 times as long as apical width. Ovipositor sheath longer than fore wing. Metasomal tergites tinged with red. Russian Far East and Japan	*Himertosoma uchidai* Kuslitzky, 2007 (=*Himertosoma sulcata* Kuslitzky, 1995)
–	First metasomal tergite 1.0 times as long as apical width. Ovipositor sheath shorter than fore wing. Metasomal tergites black, without conspicuous reddish areas. Egypt, Israel, Syria and Uzbekistan	*Himertosoma superbum* Schmiedeknecht, 1900
3	Propodeum covered with transverse striations in front of posterior transverse carina. Malar space 0.7 times as long as basal width of mandible. Ovipositor very long, its sheath 3.6 times as long as hind tibia. The Philippines	*Himertosoma townesi* Chandra & Gupta, 1977
–	Propodeum nearly entirely covered with punctures before posterior transverse carina (Fig. 6). Malar space and ovipositor sheath not as above	4
4	Mesosoma bicoloured (black and yellow). Malar space 0.5 times as long as basal width of mandible. Ovipositor sheath 2.0 times as long as hind tibia. The Philippines	*Himertosoma philippense* Chandra & Gupta, 1977
–	Mesosoma tricoloured (black, yellow and red) (Figs 1, 2). Malar space 1.0 times as long as basal width of mandible. Ovipositor sheath 3.0 times as long as hind tibia. Japan (the Ryukyus)	*Himertosoma kuslitzkii* sp. n.

## Discussion

While faunal information about ichneumonids in the Ryukyus is poor, several endemic or geographically important species are known from this archipelago (e.g. Momoi 1970). Recently, Matsumoto and Broad (2011) discovered two species of the genus *Rodrigama* Gauld, which occupies a basal branch in the Poemeninae, from Okinawajima Island in the Ryukyus and from Taiwan. In addition, a species of the genus *Tossinola* Viktorov, an atrophine genus with a fragmented Old World distribution, was discovered on the Yakushima, Tokunoshima and Okinawajima Islands in the Ryukyus as well as from Taiwan (Watanabe et al. 2010; Watanabe 2012).

This archipelago is located on the border between the Palaearctic and the Oriental Regions. Some ichneumonids of the Ryukyus, including the above mentioned species, show a tendency towards having the same or closely related species distributed in adjacent areas, viz. Southern China, Taiwan and Southwestern Japan (Momoi 1970; Watanabe et al. 2010; Matsumoto and Broad 2011; Watanabe 2012). The discovery of a new species of *Himertosoma* in the Ryukyus suggests that this or closely related species should also be found in these biogeographically important areas.

## Supplementary Material

XML Treatment for Himertosoma

XML Treatment for Himertosoma kuslitzkii
